# Sfr1, a *Tetrahymena thermophila* Sfi1 Repeat Protein, Modulates the Production of Cortical Row Basal Bodies

**DOI:** 10.1128/mSphere.00257-16

**Published:** 2016-11-16

**Authors:** Westley Heydeck, Alexander J. Stemm-Wolf, Janin Knop, Christina C. Poh, Mark Winey

**Affiliations:** Department of Molecular, Cellular and Developmental Biology, University of Colorado Boulder, Boulder, Colorado, USA; University at Buffalo

**Keywords:** basal body, centrin, centriole, Sfi1 repeat, *Tetrahymena*, ciliates

## Abstract

Basal bodies are essential microtubule-based structures that template, anchor, and orient cilia at the cell surface. Cilia act primarily in the generation of directional fluid flow and sensory reception, both of which are utilized for a broad spectrum of cellular processes. Although basal bodies contribute to vital cell functions, the molecular contributors of their assembly and maintenance are poorly understood. Previous studies of the ciliate *Tetrahymena thermophila* revealed important roles for two centrin family members in basal body assembly, separation of new basal bodies, and stability. Here, we characterize the basal body function of a centrin-binding protein, Sfr1, in *Tetrahymena*. Sfr1 is part of a large family of 13 proteins in *Tetrahymena* that contain Sfi1 repeats (SFRs), a motif originally identified in *Saccharomyces cerevisiae* Sfi1 that binds centrin. Sfr1 is the only SFR protein in *Tetrahymena* that localizes to all cortical row and oral apparatus basal bodies. In addition, Sfr1 resides predominantly at the microtubule scaffold from the proximal cartwheel to the distal transition zone. Complete genomic knockout of *SFR1* (*sfr1*Δ) causes a significant increase in both cortical row basal body density and the number of cortical rows, contributing to an overall overproduction of basal bodies. Reintroduction of Sfr1 into *sfr1*Δ mutant cells leads to a marked reduction of cortical row basal body density and the total number of cortical row basal bodies. Therefore, Sfr1 directly modulates cortical row basal body production. This study reveals an inhibitory role for Sfr1, and potentially centrins, in *Tetrahymena* basal body production.

**IMPORTANCE** Basal bodies and centrioles are structurally similar and, when rendered dysfunctional as a result of improper assembly or maintenance, are associated with human diseases. Centrins are conserved and abundant components of both structures whose basal body and centriolar functions remain incompletely understood. Despite the extensive study of centrins in *Tetrahymena thermophila*, little is known about how centrin-binding proteins contribute to centrin’s roles in basal body assembly, stability, and orientation. The sole previous study of the large centrin-binding protein family in *Tetrahymena* revealed a role for Sfr13 in the stabilization and separation of basal bodies. In this study, we found that Sfr1 localizes to all *Tetrahymena* basal bodies and complete genetic deletion of *SFR1* leads to overproduction of basal bodies. The uncovered inhibitory role of Sfr1 in basal body production suggests that centrin-binding proteins, as well as centrins, may influence basal body number both positively and negatively.

## Abstract (precis)

Basal bodies and centrioles are structurally similar and, when rendered dysfunctional as a result of improper assembly or maintenance, are associated with human diseases. Centrins are conserved and abundant components of both structures whose basal body and centriolar functions remain incompletely understood. Despite the extensive study of centrins in *Tetrahymena thermophila*, little is known about how centrin-binding proteins contribute to centrin’s roles in basal body assembly, stability, and orientation. The sole previous study of the large centrin-binding protein family in *Tetrahymena* revealed a role for Sfr13 in the stabilization and separation of basal bodies. In this study, we found that Sfr1 localizes to all *Tetrahymena* basal bodies and complete genetic deletion of *SFR1* leads to overproduction of basal bodies. The uncovered inhibitory role of Sfr1 in basal body production suggests that centrin-binding proteins, as well as centrins, may influence basal body number both positively and negatively.

## INTRODUCTION

Microtubule-organizing centers (MTOCs) are widely conserved eukaryotic structures that nucleate microtubules for cellular control of vital processes, including centriole-comprised centrosomes assembling and organizing the mitotic spindle during cell division and basal bodies anchoring and templating cilium formation at the cell surface. Although basal bodies and centrioles serve distinct functions, they are structurally related, sharing many components, and are interconverted in multiple cell types (1–3). Centrosomal amplification is observed in tumor cells and can lead to both increased genomic instability and tumor aggressiveness (4). Most human cells are ciliated and require proper basal body function to form primary or motile cilia, which act predominantly in sensory reception and generation of directional fluid flow, respectively (5). A class of human diseases known as ciliopathies is associated with underlying genetic mutations in both basal body and ciliary components, giving rise to a diverse set of disease manifestations (6–8).

Basal bodies are cylindrical, microtubule-based structures that typically display a 9-fold radially symmetric array of triplet microtubules and are organized structurally into three distinct domains (9). The proximal region contains the cartwheel structure encompassing nine radial spokes emanating from a central hub to the microtubule triplets. At the distal end is the transition zone or the region of transition from the microtubule triplets of the basal body to the microtubule doublets of the ciliary axoneme. Third, the central core, or midpoint, lies between the proximal and distal regions of the basal body. The specialized microtubules that comprise basal bodies lend a unique, hyperstabilized property allowing this structure to remain functional through multiple cell divisions, compared to the dynamic microtubules that perform other essential cellular functions (10). Despite the importance of basal bodies and centrioles, the fundamental mechanisms for the assembly and maintenance of these structures, especially on a molecular level, remain poorly understood.

New basal body and centriole assembly is tightly regulated for specific cellular requirements, with assembly either limited to one site on an existing structure or occurring through an amplification process of mass assembly (11). Assembly generally occurs from an extant structure in a 1:1 ratio with the aid of molecular assembly factors (12–15). Additionally, basal bodies and centrioles can be assembled *de novo*, as well as from a deuterosome serving to mediate the amplification of these structures in specific cell types (16–18). Upon assembly, basal bodies and centrioles are stabilized by posttranslational tubulin modifications, as well as molecular stability factors (10, 19–23). The lack of mechanistic detail pertaining to the assembly and stability of basal bodies has prompted the employment of multiple approaches to identify additional molecular components critical to these processes (9, 24, 25).

One such molecular component is centrin, a widely conserved calcium-binding protein that ubiquitously associates with MTOCs, including basal bodies and centrioles (9, 24–26). Functional analyses of centrin in MTOCs identified important roles in yeast spindle pole body (SPB, yeast centrosome) duplication, basal body assembly (including properly orienting new basal bodies), basal body separation, and basal body stability (21, 27–31). Furthermore, studies of mammalian centrin have identified an additional requirement for centrin in the formation of both motile and primary cilia (32–34). Aside from functional roles in MTOCs, centrins have been implicated in other processes, including DNA damage repair, nuclear export, and regulation of fibroblast growth factor-mediated signaling (35–38).

Given the multifaceted functions of centrin, it is thought that temporal and spatial constraints on centrin are provided by centrin-binding partners. Previous studies revealed an interaction between *Saccharomyces cerevisiae* centrin (Cdc31) and an alpha-helical protein, Sfi1, at the yeast SPB, where both proteins are essential for SPB duplication (27, 39, 40). A hydrophobic pocket in centrin’s C-terminal domain binds an internal sequence repeat within Sfi1 with a 33-amino-acid periodicity (yeast Sfi1 contains 21 repeats), now known as an Sfi1 repeat (SFR) or centrin-binding repeat. Each repeat has the capacity to bind one centrin molecule and contains an embedded, conserved sequence motif, Ax7LLx3F/Lx2WK/R, that is required for this interaction (39, 40). Sfi1 is the only protein in the yeast genome that contains SFRs. Utilizing this sequence motif information obtained from yeast Sfi1, centrin-binding proteins have been uncovered in ciliates and vertebrates, highlighting a similarly high degree of conservation in eukaryotes between centrin and its binding partners (39, 41–44). Through a search of the human genome, humans appear to have five proteins with SFRs, of which two (Poc5 and Sfi1) have been studied (39, 43). Notably, both human Sfi1 and human Poc5 (hPoc5) localize to centrioles (39, 43). In addition, hPoc5 was found to bind hCetn2 and hCetn3 through its SFRs and is required for centriole elongation/maturation (43). In *Paramecium tetraurelia*, centrin-binding proteins have an important role in maintaining the structural integrity of the centrin-rich cytoskeleton, called the infraciliary lattice (41, 42).

The free-swimming ciliate *Tetrahymena thermophila* is a powerful system for studying basal body dynamics because it has a multitude of basal bodies, organized in cortical rows running along the anterior-posterior axis of the cell, as well as a basal body-comprised feeding structure known as the oral apparatus (45). Importantly, *Tetrahymena* basal bodies share structural similarities and conserved molecular components with humans (9, 46, 47). Previous studies have identified and characterized important molecular contributors to basal body dynamics, including the function of Cen1 (human Cetn2 homologue) in basal body assembly and maintenance and Cen2 (human Cetn3 homologue) in basal body maintenance and orientation (21, 30, 31). Furthermore, a large family of 13 SFR proteins, named Sfr1 to Sfr13, was discovered by mining the *Tetrahymena* genome for proteins containing the SFR conserved sequence motif (44). Interestingly, localization studies revealed that the majority of these proteins localize to basal bodies and reside in distinct domains that heavily overlap centrin localization (21, 30, 31, 44). Functional studies have previously been conducted with only a single member of this family, Sfr13, which was shown to bind centrin and have an important role in separating and stabilizing *Tetrahymena* basal bodies (44).

In this study, we investigate the functional role of Sfr1, a member of the centrin-binding protein family in *Tetrahymena*. Sfr1 was found to localize to all *Tetrahymena* basal bodies, more specifically, primarily along the microtubule scaffold. Upon the generation of a complete genomic *SFR1* knockout, *Tetrahymena* cells overproduced basal bodies, resulting in increased basal body density along cortical rows.

## RESULTS

### Sequence confirmation of *T. thermophila* Sfr1 and homology with *P. tetraurelia* Sfr1.

A previous study identified a large family of 13 SFR proteins (Sfr1 to Sfr13) in the *Tetrahymena* genome, of which 9 were reported to localize to basal bodies (44). Through image averaging of cortical row basal bodies, it was shown that these 9 SFR proteins localize asymmetrically around the basal body periphery, except for Sfr1, which exhibits no asymmetry relative to the basal body and resides at the basal body proper. The unique basal body location of Sfr1 relative to other SFR family members suggests a nonredundant basal body function(s) and prompted further investigation of the role of Sfr1 in basal bodies.

**FIG 1:**
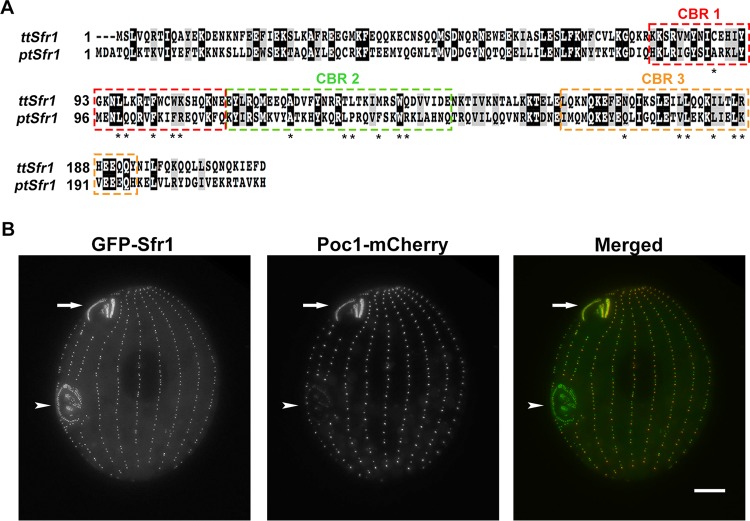
Sfr1 localizes to all basal bodies in *T. thermophila*. (A) Sequence alignment of Sfr1 homologues in two ciliates, *T. thermophila* (TTHERM_00463380) and *P. tetraurelia* (GSPATP00030126001). Both homologues contain three SFRs or centrin-binding repeats (CBRs) highlighted in colored, dashed boxes. CBR1 and CBR2 are oriented in tandem, while CBR3 is isolated in the C terminus. Identical residues are shaded in black, and similar residues are shaded in gray. Residues marked with asterisks denote the conserved sequence motif found within CBRs (Ax7LLx3F/Lx2WK/R). Sequence comparison of both Sfr1 homologues: overall identities, 55/194 (28%); positives, 108/194 (55%). (B) Live-cell imaging of N-terminally tagged MTT1pr-GFP-Sfr1 colocalized with C-terminally tagged Poc1-mCherry (44). MTT1pr-GFP-Sfr1 localizes to all cortical row basal bodies, the mature oral apparatus (arrows), and the developing oral apparatus (arrowheads). Scale bar, 10 µm.

Microarray expression data available in the *Tetrahymena* Genome Database indicate that Sfr1 is the most highly expressed SFR protein during logarithmic growth (48). To confirm the cDNA sequence of Sfr1, RNA was isolated from wild-type (WT) *Tetrahymena* cells and reverse transcription (RT)-PCR revealed a cDNA sequence containing a previously unannotated 88-bp intron in the N terminus (N terminal to the SFRs). This intronic sequence was not annotated in the *Tetrahymena* Genome Database, and as a result, the confirmed cDNA sequence of Sfr1 was not used in the previous study of *Tetrahymena* SFR proteins (44). Importantly, the previous annotation of Sfr1 was still in frame but exhibited a different 5′ end. Sfr1 is 212 amino acids in length (27.8 kDa) and contains three SFRs, organized as a tandem repeat (similar to hPoc5 [43]) and as an isolated C-terminal repeat (Fig. 1A). Interestingly, the Basic Local Alignment Search Tool (BLAST) revealed that Sfr1 is restricted to only a subset of ciliates, *T. thermophila* and *P. tetraurelia*. The *Paramecium* Sfr1 homologue, derived from a reciprocal best BLAST search, shares SFR organization and amino acid sequence homology both within and outside the SFRs (overall identities, 55/194 [28%]; positives, 108/194 [55%]) (49).

### Sfr1 localizes to all *Tetrahymena* basal bodies.

In order to observe the localization of Sfr1 by live-cell analysis, the confirmed Sfr1 sequence was N terminally tagged with green fluorescent protein (GFP), cloned into the exogenous RPL29 locus through homologous recombination, and placed under the control of the cadmium-inducible *MTT1* promoter (50, 51). *MTT1*pr-GFP-Sfr1 was then transformed into cells endogenously expressing a known basal body marker, Poc1-mCherry (10). Overexpressed Sfr1 colocalizes with Poc1 in all basal bodies along the highly organized cortical rows and in both the immature and mature basal-body-comprised feeding structures of the cell, the oral apparatus (Fig. 1B). Of note, endogenous Poc1 exhibits an uneven expression pattern along cortical rows, reflecting a previously reported slow incorporation into newly assembled basal bodies and gradual accumulation in maturing basal bodies (10). Similar to the previous analysis of SFR proteins in *Tetrahymena*, Sfr1 could not be endogenously tagged; therefore, all analysis was conducted with *MTT1*pr-GFP-Sfr1 (44).

### Sfr1 localizes broadly throughout the basal body microtubule scaffold.

**FIG 2:**
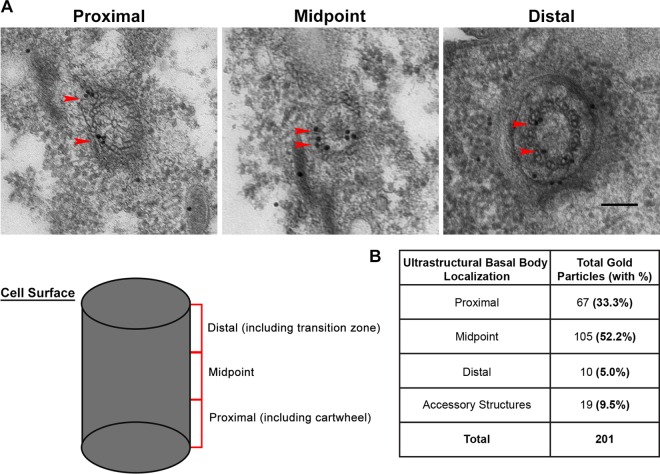
Sfr1 localizes broadly throughout the basal body microtubule scaffold. immunoEM of N-terminally tagged MTT1pr-GFP-Sfr1 cells. (A) Serial sections reveal a majority of Sfr1 (gold particles) within the midpoint region of the basal body. Sfr1 localizes primarily along the microtubule scaffold (arrowheads). Scale bar, 100 nm. The schematized basal body indicates the proximal, midpoint, and distal regions used for quantification in panel B. (B) Quantification of the ultrastructural basal body localization of Sfr1 is based on data from seven complete serial sections from three different cells (a total of 201 gold particles). Sfr1 localizes primarily to the proximal and midpoint regions (85.5% total), with a small population at the distal end (5%) and peripheral to the basal body along the accessory structures (9.5%).

Precise basal body localization was determined by immunoelectron microscopy (immunoEM) with *MTT1*pr-GFP-Sfr1 cells and a gold-conjugated antibody against GFP. As shown in serial transverse sections from a cortical row basal body, Sfr1 localizes primarily to the microtubules extending from the proximal cartwheel structure to the distal transition zone of the basal body (red arrowheads in Fig. 2A).

Through comprehensive analysis of seven complete serials from three different *MTT1*pr-GFP-Sfr1 cells, the ultrastructural basal body localization was quantified within known basal body domains (Fig. 2B). Sfr1 is concentrated at the midpoint of the basal body (52.2%) and at the proximal cartwheel (33.3%), with a smaller population found at the distal transition zone (5%). Only 9.5% of Sfr1 was detected in the basal body periphery, often adjacent to basal body accessory structures that aid in properly orienting basal bodies along cortical rows (13). The ultrastructural basal body localization of Sfr1 partially overlaps that of centrin, which was previously found to be enriched in the microtubules at the basal body midpoint and at the distal transition zone but also resides at the site of new basal body assembly, where Sfr1 is notably absent (9, 30). Enriched localization of Sfr1 primarily along the microtubule scaffold suggested a role for Sfr1 in basal body assembly and/or maintenance rather than the modulation of basal body orientation along cortical rows. It is thought that other SFR proteins that localize to accessory structures, particularly the kinetodesmal fiber, function to properly orient basal bodies (47).

### Generation of complete genomic knockout of *SFR1.*

**FIG 3:**
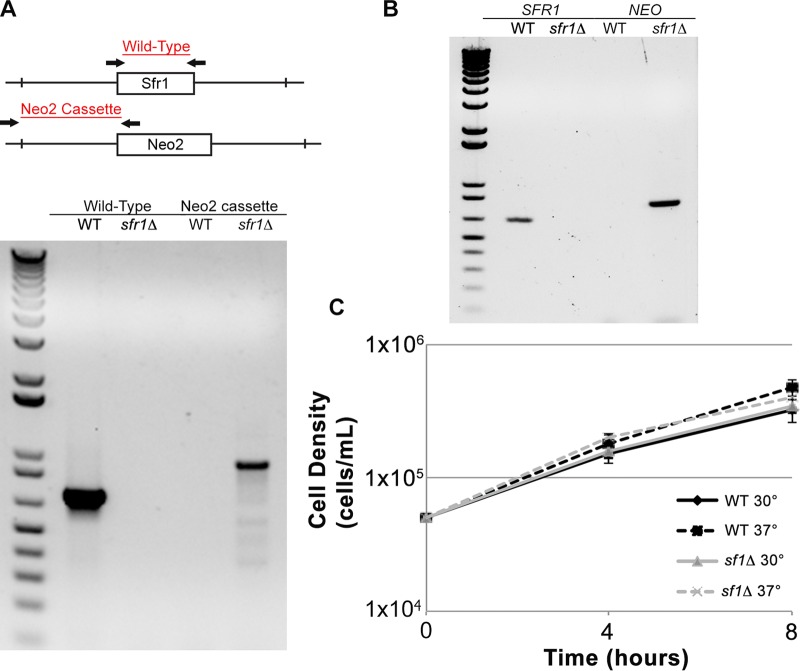
*sfr1*Δ mutant cells are viable and do not exhibit growth defects. Complete micronuclear knockout of SFR1 was performed. (A) PCR confirming complete micronuclear knockout of SFR1. The schematic depicts the homologous recombination strategy employed to generate *sfr1*Δ mutant cells. The arrows indicate the primers used to amplify both WT and deletion-specific PCR products. Only WT cells contain the SFR1 ORF, and only *sfr1*Δ mutant cells contain the Neo2 cassette. (B) RT-PCR analysis confirmed the knockout of SFR1. Expression of *SFR1* was seen only in WT cells, while only *sfr1*Δ mutant cells expressed *NEO*. (C) Logarithmic growth curves for WT and *sfr1*Δ mutant cells grown in SPP medium at either 30°C or 37°C for 8 h. Cell density measurements were taken at the initiation of the growth experiment, as well as 4 and 8 h after initiation. *n* = 2 samples per analyzed strain and growth temperature. Error bars indicate standard deviations.

To determine the function(s) of Sfr1, a complete genomic knockout strain was generated by replacing the *SFR1* open reading frame (ORF) with a Neo2 cassette conferring paromomycin resistance for selection. Knockout cells were confirmed by PCR with two oligonucleotide pairs. One pair (WT) was designed to amplify a sequence specific to the WT *SFR1* ORF, whereas the second pair (Neo cassette) was designed to amplify a sequence specific to the Neo2 cassette in order to detect the presence of the knockout construct (Fig. 3A). Isolated genomic DNA from *sfr1*Δ mutant cells showed a robust PCR product from the Neo cassette amplification but no evidence of WT *SFR1*. RNA was also isolated from WT and *sfr1*Δ mutant cells in order to perform RT-PCR analysis to confirm that *sfr1*Δ mutant cells completely lacked *SFR1* (Fig. 3B). RT-PCR validated the complete genomic knockout strain, revealing expression of *SFR1* only in WT cells and expression of *NEO* only in *sfr1*Δ mutant cells.

Cells lacking *SFR1* (*sfr1*Δ mutant cells) are viable and persist indefinitely; therefore, *SFR1* is not an essential gene for *Tetrahymena* viability. Time course analysis of the growth rates of *sfr1*Δ mutant and WT cells was conducted at both the optimal growth temperature for *Tetrahymena* (30°C) and at a temperature typically used to identify temperature-sensitive mutants (37°C) (Fig. 3C). WT and *sfr1*Δ mutant cells had similar growth rates at both temperatures.

### Loss of Sfr1 leads to increased basal body production along cortical rows.

**FIG 4:**
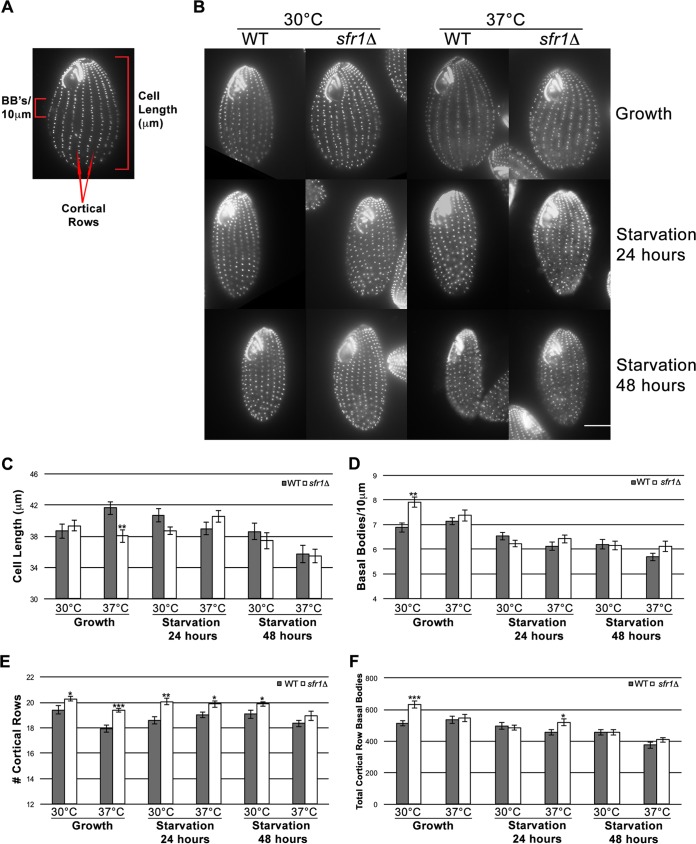
Loss of Sfr1 leads to overproduction of basal bodies along cortical rows. *sfr1*Δ mutant cells maintain a highly organized cytoskeleton with proper orientation of cortical rows but overproduce basal bodies. (A) Representative *Tetrahymena* cell fixed and stained with Cen1 antibody to label basal bodies. Three cellular parameters were measured for phenotypic analysis: cell length (in micrometers), basal body density (number of basal bodies/10 µm), and the number of cortical rows. The total number of cortical row basal bodies is derived from the equation cell length × basal body density × number of cortical rows. (B) Centrin staining of WT and *sfr1*Δ mutant cells under growth and starvation conditions. Phenotypic analysis was conducted at both 30°C and 37°C. Scale bar, 10 µm. (C to F) Quantification from phenotypic analysis of effect of loss of Sfr1 on cellular parameters. For all measured parameters, gray bars show the results from WT cells and white bars show those from *sfr1*Δ mutant cells. *n* = 15 per strain per condition. Error bars indicate the standard errors of the mean values. *P* values were derived from a Student *t* test calculation. *, *P* < 0.05; **, *P* < 0.01; ***, *P* < 0.001.

To investigate potential basal body functions of Sfr1, phenotypic analysis of growing and starved cells (starved for 24 or 48 h) was conducted at both 30°C and 37°C. WT and *sfr1*Δ mutant cells were examined for three major cellular parameters: cell length (micrometers), basal body density along cortical rows (number of basal bodies/10 µm), and the total number of cortical rows (Fig. 4A). From these measurements, an estimate of the total number of cortical row basal bodies was generated. These parameters are influenced by the ability of *Tetrahymena* cells to properly assemble and maintain basal bodies, both of which are critical processes for sustaining the spatial organization of cortical rows. Cells analyzed under growth conditions were assayed for the capacity to assemble basal bodies, while starvation conditions prevent new basal body assembly and exacerbate any existing underlying deficiencies in basal body maintenance/stability (10).

Under all of the experimental conditions used, WT and *sfr1*Δ mutant cells properly oriented basal bodies along cortical rows and maintained the correct gross morphology of their oral apparatus (Fig. 4B). Given the fact that Sfr1 resides primarily along the basal body scaffold (Fig. 2A), it was not unexpected that *sfr1*Δ mutant cells would maintain appropriate basal body orientation. As shown in Fig. 4B and quantified in Fig. 4C, differences in cell length were observed only during growth at 37°C, when *sfr1*Δ mutant cells were significantly shorter than WT cells. This indicates that deletion of *SFR1* may lead to altered cell length at a higher growth temperature but not as a result of aberrant basal body orientation (Fig. 4B).

Although loss of Sfr1 does not seem to have a large effect on cell length, *sfr1*Δ mutant cells grown at 30°C (but not at 37°C) had a significantly greater basal body density along cortical rows (7.91 basal bodies/10 µm) than WT cells (6.98 basal bodies/10 µm) (Fig. 4B and D). Even though *sfr1*Δ mutant cells assemble more basal bodies during growth, excess basal bodies are not maintained, as *sfr1*Δ mutant cells have a basal body density and a total number of basal bodies similar to those of WT cells after starvation (Fig. 4F). In addition to increased basal body density, loss of Sfr1 leads to an increase in the number of cortical rows during both growth and starvation (Fig. 4E). This increase in the number of cortical rows likely accommodates the excess basal bodies produced during growth. When basal body numbers return to normal in starvation, it may take a longer time for the cell to reorganize its cortex such that the number of cortical rows reverts to WT levels (52). Overproduction of basal bodies upon depletion of *SFR1* is a surprising result because it suggests an antagonistic role for Sfr1 in the production of basal bodies.

### Increased basal body production is the direct consequence of loss of Sfr1.

**FIG 5:**
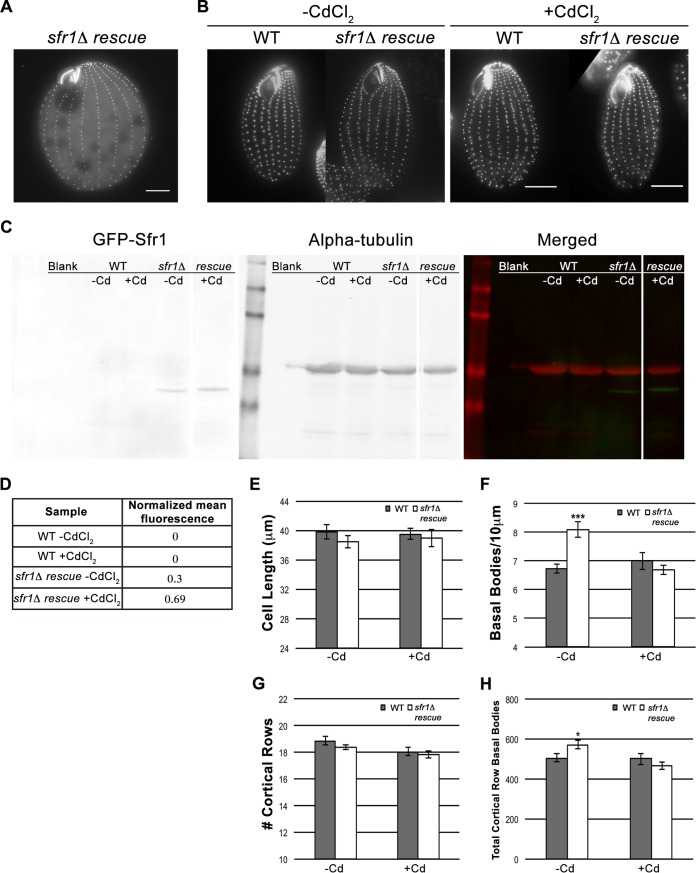
Overproduction of basal bodies is the direct consequence of loss of Sfr1. Rescue of increased basal body density and the total number of cortical row basal bodies observed in *sfr1*Δ mutant cells through the reintroduction of MTT1pr-GFP-Sfr1 (*sfr1*Δ rescue strain). (A) Analysis of live *sfr1*Δ rescue cells showing proper localization of MTT1pr-GFP-Sfr1 in the absence of SFR1 (compared with localization in WT cells containing SFR1 in Fig. 1B). (B) Centrin staining of WT and *sfr1*Δ rescue cells after overnight induction of GFP-Sfr1 (plus CdCl_2_ at 0.05 µg/ml) or in the absence of CdCl_2_. Scale bar, 10 µm. (C) Western blot analysis of both WT and *sfr1*Δ rescue cells minus CdCl_2_ and plus CdCl_2_. Antibodies against GFP (for GFP-Sfr1) and α-tubulin (as a loading control) were used. On the left are levels of GFP-Sfr1 expression, in the middle is α-tubulin expression, and on the right is a merged image of both channels. Of note, the *sfr1*Δ rescue minus Cd and plus Cd lanes are from different areas of the same immunoblot, as represented by the spacing between these lanes. (D) Normalized mean fluorescence was calculated for both the WT and *sfr1*Δ rescue strains in the presence or absence of CdCl_2_. (E to H) Quantification of the rescuing effect of induced GFP-Sfr1 expression on cells lacking Sfr1. For all measured parameters, gray bars show the results from WT cells and white bars show those from *sfr1*Δ rescue cells. *n* = 15 per strain per condition. Error bars indicate the standard errors of the mean values. *P* values were derived from a Student *t* test calculation: *, *P* < 0.05; **, *P* < 0.01; ***, *P* < 0.001.

In order to determine that the observed increase in cortical row basal body density was a direct result of the loss of Sfr1, a strain was generated by reintroducing *SFR1* into *sfr1*Δ mutant cells (*sfr1*Δ rescue strain). The *sfr1*Δ rescue strain was made by transforming a cadmium chloride (CdCl_2_)-inducible rescuing construct, *MTT1pr-*GFP-Sfr1, into the *SFR1* null strain. This approach resulted in the proper localization of GFP-Sfr1 (compared with Fig. 1B) to all cortical row and oral apparatus basal bodies without the presence of endogenous Sfr1 (Fig. 5A). To assess rescue, cell length, basal body density, and the number of cortical rows were analyzed at 30°C with CdCl_2_ (plus CdCl_2_) and without CdCl_2_ (minus CdCl_2_). An immunoblot assay verified that GFP-Sfr1 was expressed in the presence of CdCl_2_ and at a higher level than without CdCl_2_ (Fig. 5C). Importantly, GFP-Sfr1 expression was not observed in WT cells and was seen only in *sfr1*Δ rescue cells. Because of the leakiness of the *MTT1* promoter, GFP-Sfr1 was expressed at a detectable level without the addition of CdCl_2_ but at roughly half of the level (based on normalized mean fluorescence) observed with CdCl_2_ (Fig. 5D). Similar to this analysis, leaky expression of the *MTT1* promoter after depletion of CdCl_2_ has been observed previously (53).

As shown in Fig. 5B, *sfr1*Δ rescue cells maintained cortical basal body organization and proper orientation along basal body rows upon treatment with CdCl_2_. When WT and *sfr1*Δ rescue cells were incubated with CdCl_2_, cell length was also unaffected (Fig. 5E). This was not a surprising result since cell length did not differ between WT and *sfr1*Δ mutant cells grown at 30°C (Fig. 4C). Unlike cell length, cortical row basal body density was significantly higher in *sfr1*Δ rescue cells incubated without CdCl_2_, and this density decreased to WT levels when GFP-Sfr1 expression was induced with CdCl_2_ (Fig. 5F). This increase in basal body density, despite leaky expression of GFP-Sfr1, suggests that the level of expression without CdCl_2_ was below a potential threshold needed to drive changes in basal body production. Of note, the increased basal body density seen in *sfr1*Δ rescue cells without CdCl_2_ was similar to that observed in *sfr1*Δ mutant cells (Fig. 4D). Since the presence of CdCl_2_ did not have any effect on basal body density in WT cells, Sfr1 modulates basal body production along cortical rows.

Interestingly, although the *sfr1*Δ rescue strain, when uninduced, had increased basal body density, like the *sfr1*Δ mutant strain, it did not have additional cortical rows (Fig. 5G). This could be due to leaky expression from the rescuing construct, thus limiting the amount of overduplication normally observed in the null strain and circumventing the cell’s need to add cortical rows to accommodate extra basal bodies. Lastly, *sfr1*Δ rescue cells grown without CdCl_2_ had a significantly higher total number of cortical row basal bodies, as was seen in *sfr1*Δ mutant cells (Fig. 4F and 5H). When CdCl_2_ was added, the total number of cortical row basal bodies decreased to WT levels; therefore, Sfr1 also regulates the total number of cortical row basal bodies (Fig. 5H). This increase in the total number of cortical row basal bodies was a direct result of increased basal body density since the number of cortical rows did not differ between WT and *sfr1*Δ rescue cells (Fig. 5G). Collectively, the results of this rescue experiment demonstrate that Sfr1 is directly responsible for limiting the number of basal bodies in *Tetrahymena*.

## DISCUSSION

*Tetrahymena* has a highly organized cytoskeleton that requires proper assembly, orientation, and maintenance of basal bodies along cortical rows, all of which are critical processes that rely on centrin (9, 30, 31). It is largely unclear how centrin performs multiple, essential basal body functions that require precise timing and distinct centrin populations at basal body subdomains, but it is thought to be through interacting partners (9, 30, 31). Intriguingly, *Tetrahymena* has a large family of centrin-binding proteins (Sfr1 to Sfr13) that may individually be responsible for subsets of overall centrin function and collectively provide both the necessary spatial and temporal regulation of centrin at basal bodies (44). This potential spatial regulation of centrin is reflective of the previous finding that SFR family members are each restricted to distinct regions of the basal body periphery (except for Sfr1 at the basal body proper) coinciding with regions known to also contain centrin (44).

Sfr1 is the only SFR family member that localizes to the basal body microtubule scaffold, indicating a potentially nonredundant basal body function for Sfr1. The basal body localization of Sfr1 was further dissected at the ultrastructural level by immunoEM, which revealed a high percentage of Sfr1 broadly residing along the basal body microtubule scaffold from the proximal cartwheel to the distal transition zone. The ultrastructural localization of Sfr1 overlaps that of *Tetrahymena* Cen1 and Cen2 at the transition zone, and both Sfr1 and Cen1 are heavily enriched at the basal body midpoint (9, 30, 31). Aside from localization along the microtubule scaffold, Cen1 and Cen2 predominantly localize to the site of new basal body assembly, where Sfr1 is largely absent. Interestingly, previous ultrastructural analysis of Sfr13 shows that it differs from Sfr1 in being highly enriched at the site of new basal body assembly, supporting the hypothesis that centrin-binding proteins may be guiding the spatial positioning of centrin (44).

*Tetrahymena* cells lacking *SFR1* (*sfr1*Δ mutant cells) are viable and display a marked overproduction of basal bodies along cortical rows. The observed overproduction of basal bodies upon the loss of Sfr1 was rescued by reintroducing Sfr1 under the control of an inducible promoter, thus revealing a direct role for Sfr1 in the modulation of basal body production along cortical rows. *Tetrahymena* cells are strikingly capable of maintaining a nearly constant number of cortical row basal bodies by adjusting basal body density relative to the number of cortical rows (52, 54–58). Basal body number constancy has been shown to be regulated on the basis of the cellular corticotype (the number of cortical rows). The total basal body number in elevated corticotypes is balanced by a decreased basal body density, and conversely, lower corticotypes exhibit increased basal body density. Furthermore, corticotype maintenance was initially documented to be around a “stability center” at 19 cortical rows but was later refined to be around a “stability range” of 18 to 20 rows (55, 57). It is unknown what mechanisms or signaling cues underlie the ability of *Tetrahymena* cells to regulate the overall basal body number and how they direct the distribution of basal bodies to compensate for aberrant basal body density or an abnormal number of cortical rows. In the absence of Sfr1, *sfr1*Δ mutant cells unexpectedly lost this compensatory mechanism for maintaining constancy in the cortical row basal body number during logarithmic growth and instead exhibited both increased basal body density and an increased number of cortical rows relative to WT cells (and therefore a significant increase in the total number of basal bodies). Although *sfr1*Δ mutant cells overproduce basal bodies during growth, the condition of having excess basal bodies does not appear to be stable, as starved *sfr1*Δ mutant cells have a total number of cortical row basal bodies similar to that of WT cells. It is not yet understood if this function of Sfr1 is unique or if other SFR family members have an inhibitory effect on the basal body number.

Similar to the basal body number constancy observed in *Tetrahymena*, the centriole number was also found to be intrinsically constant in some cancer cell lines (59). Cancer cell lines with supernumerary centrioles treated with a specific inhibitor of the centriole assembly regulator Polo-like kinase 4 (Plk4) showed a reduction in centriole number due to cell division in the absence of centriole assembly. Upon removal of the inhibitor, the centriole number reverted back to the previous supernumerary state. This suggests the existence of a mechanism in cells that maintains a set number of basal bodies and centrioles—a mechanism that may be conserved across eukaryotes. More research is needed to address the molecular underpinnings of this process.

Centrins and their interacting proteins are good candidates for participants in such a process. The role of Sfr1 in the modulation of basal body production does not correspond to reported functions of centrins in *Tetrahymena*. This could be because Sfr1 acts independently of centrin or because this role for centrin may have been undetectable in previous studies of centrin-null cells that display the full effects of centrin loss (21, 30, 31). Intriguingly, it has recently been shown that human Cetn3 can antagonize human Cetn2 function in centriole assembly, highlighting a role for Cetn3 in the inhibition of centrosome duplication (60). Depletion of Cetn3 in HeLa cells resulted in the overproduction of centrioles, providing an exciting potential parallel to our observed overproduction of basal bodies upon loss of the centrin-binding protein Sfr1. Further analysis of centrin-binding proteins is needed to understand their contribution to known centrin functions and how specificity of binding to the two functionally distinct centrin families is attained. Given evidence that centrins can act antagonistically in regards to centriole assembly, centrin-binding proteins may act to bidirectionally modulate basal body production and therefore the basal body number through interaction with a specific centrin.

## MATERIALS AND METHODS

### *T. thermophila* strains and culture media.

The WT strain used as a control comparison to assay growth rates and for phenotypic analysis of the *sfr1*Δ mutant and *sfr1*Δ rescue strains was derived from the progeny of a cross between B2086 and CU428 (each parental strain from the *Tetrahymena* Stock Center, Cornell University, Ithaca, NY). Cells were grown in 2% super proteose peptone (SPP) medium (61) at 30°C for localization experiments. For phenotypic analysis of the *sfr1*Δ mutant strain, cells were grown in EPP medium (62) at either 30°C or 37°C as described. For phenotypic analysis of the *sfr1*Δ mutant strain during starvation, cells were grown overnight in EPP medium to mid-log density (cell density was measured with a Z2 Coulter Counter [Beckman Coulter, Inc.]) at 30°C or 37°C, washed with 10 mM Tris-HCl (pH 7.4), and resuspended in 10 mM Tris-HCl (pH 7.4) for either 24 or 48 h at 30°C or 37°C. Analysis of the *sfr1*Δ rescue strain was performed by growing cells overnight in EPP medium and starving them in 10 mM Tris-HCl (pH 7.4) for 5 h (to eliminate dividing cells) before fixation. EPP medium was used for all phenotypic analysis for consistency between analyses and because EPP medium lacks a metal chelator; therefore, less CdCl_2_ was need for induction.

### cDNA synthesis and sequence comparison of Sfr1 homologues.

*Tetrahymena SFR1* (TTHERM_00463380) cDNA was generated with the Superscript II One-Step RT-PCR system (Invitrogen) and then cloned into the pBluescript KS(−) construct for sequencing. The confirmed cDNA sequence of *SFR1* was logged at the *Tetrahymena* Genome Database Wiki, and the gene information is searchable by name (“Sfr1”) in addition to its TTHERM identifier. The *Tetrahymena* Sfr1 protein sequence was used as the query sequence for a BLAST search of the *P. tetraurelia* genome (*Paramecium* Genome Database [ParameciumDB]) to uncover the *Paramecium* Sfr1 homologue (GSPATP00030126001), which serves as the reciprocal best BLAST hit. Multiple-sequence alignment of the two Sfr1 homologues and box shading of residues were performed with ClustalW and BoxShade, respectively (ExPASy).

### Plasmids and strain construction.

The exogenous N-terminal GFP fusion used to localize Sfr1 was generated by cloning *SFR1* first into the pENTR4 Dual Selection Vector (Invitrogen) and then subcloning it into pBS-*MTT1*-GFP-gtw (51) by utilizing the Gateway cloning system (Invitrogen). This resulted in N-terminally tagged GFP-Sfr1, under the control of the *MTT1*-inducible promoter, at the RPL29 locus (conferring cycloheximide resistance). Exogenous Sfr1 was coexpressed with endogenous Poc1 (10) tagged with mCherry at its C terminus for colocalization studies. Macronuclear transformation of both DNA constructs into *T. thermophila* was performed with a PDS-1000 particle bombarder (Bio-Rad) in accordance with previous studies (63). For all experiments with the inducible *MTT1* promoter, constructs were induced by incubating cells overnight in CdCl_2_ (0.5 µg/ml for SPP medium and 0.05 µg/ml for EPP medium).

### **Generation of** ***sfr1***Δ** mutant and** ***sfr1***Δ **rescue strains.**

The strains used for micronuclear transformation (as described in reference 63) were B2086/crNeo and CU428/crNeo, both of which contain two frameshift mutations in the *NEO* gene in the macronucleus to prevent DNA elimination of the selectable marker following micronuclear transformation (64, 65). The construct used to delete *SFR1* contains the Neo2 cassette along with 1 kb of flanking sequence upstream of the start codon and 1 kb of flanking sequence downstream of the stop codon (66). DNA was used to coat gold particles, and biolistic bombardment of conjugating cells (with the Bio-Rad PDS-1000) was conducted. Cells were allowed to finish conjugation after bombardment by being maintained in 10 mM Tris-HCl (pH 7.4) overnight before transfer into SPP medium for ensuing drug selection. Integration into the *SFR1* locus was confirmed by PCR. Micronuclear knockout heterokaryons of two different mating types were generated with star strains (67). Heterokaryons were mated to eliminate *SFR1* from both nuclei (somatic and germline), creating *sfr1*Δ mutant cells. Deletion of *SFR1* and replacement with the Neo2 cassette were validated by PCR and RT-PCR with WT *SFR1* forward primer TAAGTTTAGTTTAGAGAACAATATAGGCTTATGAGAAGG, WT *SFR1* reverse primer TCAATCAAATTCAATTTTTTAATTTTATGAAAGG, Neo cassette forward primer AATCTACTAATTTGCTTTATTTTTCATAAGC, and Neo cassette reverse primer TCCATACTTTGAAGATATCAAGC. For RT-PCR, RNA was isolated from WT and *sfr1*Δ mutant cells with the RNeasy Mini and QIAshredder kits supplied by Qiagen. cDNA was generated with the Superscript IV One-Step RT-PCR system (Invitrogen). Ensuing standard PCR used a pair of primers to detect *SFR1* (*SFR1* forward primer AAGTTTAGTTTAGAGAACAATATAGGCTTATGAGAAGG and *SFR1* reverse primer TCAATCAAATTCAATTTTTTAATTTTAATTTTATGAAAGG) and a pair to detect *NEO* (*NEO* forward primer ATGGCAAGCTTGGATGGATTGCACGC and *NEO* reverse primer TCAGAAGAACTCGTCAAGAAGGCG). The *sfr1*Δ rescue strain was made by reintroducing SFR1 into *sfr1*Δ mutant cells by biolistics in order to incorporate pBS-*MTT1*-GFP-Sfr1 into the somatic macronucleus. Transformed cells were selected by adding cycloheximide (pBS-*MTT1*-GFP-Sfr1 confers cycloheximide resistance), and *SFR1* reintroduction into the *sfr1*Δ rescue strain was through CdCl_2_ induction.

### Fluorescence imaging.

All image collection was conducted at room temperature with an Eclipse Ti inverted microscope (Nikon, Japan) with a CFI Plan Apo VC 60× H numerical aperture 1.4 objective (Nikon, Japan) and a charge-coupled device camera (CoolSNAP HQ2; Photometrics, Tucson, AZ). Image acquisition was through the MetaMorph Imaging Software (Molecular Devices, Sunnyvale, CA).

For live-cell imaging of GFP- and mCherry-tagged proteins, cells were washed in 10 mM Tris-HCl (pH 7.4), pelleted, and placed on microscope slides (VWR, Radnor, PA). For immunofluorescence assay, cells were fixed with 3% formaldehyde, followed by 15% ethanol (68). Fixed cells were placed on poly-l-lysine-coated multiwell slides (Polysciences, Warrington, PA) and blocked for 1 h with phosphate-buffered saline (PBS) plus 1% bovine serum albumin (BSA) before primary antibody incubation. The *Tetrahymena* Cen1 antibody was used at 1:2,000 (30), and antibody incubation was carried out overnight at 4°C. The secondary antibody used for a 2-h room temperature incubation was Alexa Fluor 488 (Invitrogen)-conjugated anti-rabbit antibody at 1:1,000. Primary and secondary antibodies were in PBS plus 1% BSA, and washes between antibody incubations were performed with PBS plus 0.1% BSA. Cells were mounted in CitiFluor (Ted Pella, Redding, CA).

### immunoEM.

*Tetrahymena* cells were prepared for ultrastructural localization by high-pressure freezing with a Wohlwend Compact 02 high-pressure freezer (Technotrade International, Manchester, NH), followed by freeze substitution (69, 70). Cells were freeze substituted in 0.25% glutaraldehyde and 0.1% uranyl acetate in acetone and embedded in Lowicryl HM20. immunoEM was conducted by making 70-nm-thick sections that were incubated with a rabbit polyclonal GFP antibody (generously gifted by Chad Pearson) and then a 15-nm gold-conjugated anti-rabbit secondary antibody (Ted Pella, Redding, CA). Samples were imaged with a Philips CM 100 transmission electron microscope. The location of gold particles was analyzed relative to known structural domains within basal bodies as previously described (9).

### Western blot analysis.

Cells were grown overnight at 30°C in EPP medium to mid-log density and then starved for 5 h (incubated with or without 0.05 µg/ml CdCl_2_ to induce GFP fusion). Whole-cell extracts were made by lysing ~30,000 cells (based on cell density measurements with a Z2 Coulter Counter [Beckman Coulter, Inc.]) in lysis buffer made of 50 mM Tris-HCl (pH 8.5), 150 mM NaCl, 20 mM EDTA, 1% Triton X-100, and 1% sodium deoxycholate plus protease inhibitors (0.2 mg/ml soybean trypsin inhibitor, 10 µg/ml leupeptin, 1 µg/ml aprotinin, 1 µg/ml pepstatin A, 10 µg/ml *N*_α_-*p*-tosyl-l-arginine methyl ester [TAME], and 10 µg/ml benzamidine). Six thousand cells were loaded per lane of a 10% SDS-PAGE gel and transferred to an Immobilon-P polyvinylidene difluoride membrane (EMD Millipore, Billerica, MA) with a transfer apparatus (Bio-Rad Labs, Hercules, CA). The membrane was incubated in Tris-buffered saline (TBS) plus 0.05% Tween 20 (TBST) with 1% BSA. Primary antibodies were a primary rabbit anti-*Aequorea victoria* GFP polyclonal antibody (TaKaRa Bio USA, Mountain View, CA) at 1:1,000 and a mouse anti-Atu1 monoclonal antibody (12G10; a generous gift of Joseph Frankel, University of Iowa, Iowa City, IA) at 1:2,500 (both in TBST plus 1% BSA). Secondary antibodies were anti-rabbit IR 800 and anti-mouse IR 680 antibodies, both at 1:10,000 (in TBST plus 1% BSA), and the signal was detected with a LI-COR Odyssey infrared imager (LI-COR, Lincoln, NE). The normalized mean fluorescence from split-channel acquired images was calculated by drawing a box within the GFP signal and obtaining the mean fluorescence value with ImageJ. This value was then background subtracted by using the same size box to measure the background mean fluorescence. Finally, the ratio of GFP to α-tubulin (loading control) fluorescence was used to get the final, normalized mean fluorescence values.

### Analysis of *sfr1*Δ mutant strain. (i) Calculation of growth rates.

A Z2 Coulter Counter (Beckman Coulter, Inc.) was used to measure the cell density of log-phase cultures to determine growth rates. The time course of growth was initiated with all strains set to a beginning density of 0.5 × 10^5^ cells/ml. Cells were allowed to grow for 8 h in SPP medium at 30°C or 37°C with measurements taken at 0, 4, and 8 h. The WT strain used is progeny from B2086 × CU428 (as described in the paragraph on *T. thermophila* strains and culture media).

### (ii) Quantification of cell length and cortical row basal bodies.

Measurements of cell length (in micrometers), basal body density (number of basal bodies/10 µm), and the number of cortical rows were done by ImageJ analysis of fixed cells (WT, *sfr1*Δ mutant, or *sfr1*Δ rescue) with basal bodies labeled with the Cen1 antibody. Cell length was calculated by using the line tool function on scaled images. A Z-stack of the entire cell was captured with MetaMorph Imaging Software (Molecular Devices, Sunnyvale, CA) in order to count the cortical rows in the entirety of the cell. Basal body density was determined by measuring a 10-µm region of three separate cortical rows with ImageJ, with two rows on the side containing the oral apparatus and the third row on the opposite side of the cell. The total number of cortical row basal bodies was determined by multiplying all three cellular parameters as previously described (44).
